# Supplementary material for: The causal relationship between the human gut microbiota and pyogenic arthritis: a Mendelian randomization study
Source: Front Cell Infect Microbiol. 2024 Nov 26;14:1452480. doi: 10.3389/fcimb.2024.1452480 (PMC11629706; doi:10.3389/fcimb.2024.1452480)
Supplement: Supplementary file 1 [file DataSheet1.zip › mendelian test/ebi-a-GCST90027616.csv_forest.pdf]

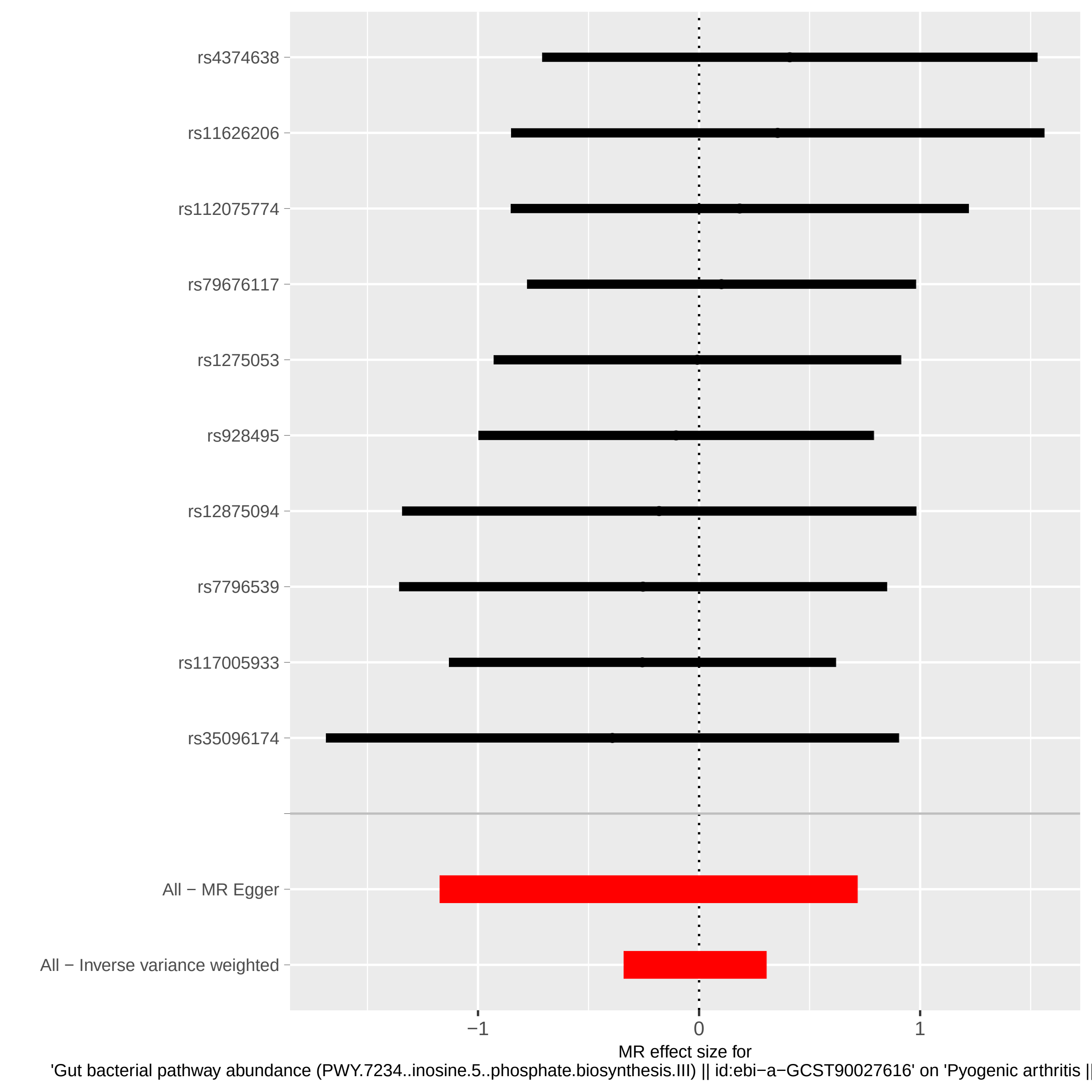
rs4374638
rs11626206
rs112075774
rs79676117
rs1275053
rs928495
rs12875094
rs7796539
rs117005933
rs35096174
All − MR Egger
All − Inverse variance weighted
−1
0
1
MR effect size for
'Gut bacterial pathway abundance (PWY.7234..inosine.5..phosphate.biosynthesis.III) || id:ebi−a−GCST90027616' on 'Pyogenic arthritis ||
